# Amorphous FeCoCrSiB Ribbons with Tailored Anisotropy for the Development of Magnetic Elements for High Frequency Applications

**DOI:** 10.3390/ma15124160

**Published:** 2022-06-12

**Authors:** Galina V. Kurlyandskaya, Luis Lezama, Anna A. Pasynkova, Stanislav O. Volchkov, Vera A. Lukshina, Aitor Larrañaga, Natalia V. Dmitrieva, Anastasia V. Timofeeva, Iñaki Orue

**Affiliations:** 1 Department of Electricity and Electronics, Basque Country University (UPV/EHU), 48940 Leioa, Spain; 2 Institute of Natural Sciences and Mathematics, Ural Federal University, 620002 Ekaterinburg, Russia; anna.chlenova@urfu.ru (A.A.P.); stanislav.volchkov@urfu.ru (S.O.V.); 3103anastasiya98@gmail.com (A.V.T.); 3 Department ofInorganic Chemistry, Basque Country University (UPV/EHU), 48940 Leioa, Spain; luis.lezama@ehu.eus; 4 Los Servicios Generales de Investigación (SGIKER), Basque Country University UPV/EHU, 48940 Leioa, Spain; aitor.larranaga@ehu.eus (A.L.); inaki.orue@ehu.eus (I.O.); 5 Laboratory of Advanced Magnetic Materials, Institute of Metal Physics UD RAS, 620108 Ekaterinburg, Russia; 6 Micromagnetism Laboratory Institute of Metal Physics UD RAS, 620108 Ekaterinburg, Russia; lukshina@imp.uran.ru (V.A.L.); dmitrieva@imp.uran.ru (N.V.D.)

**Keywords:** amorphous ribbons, magnetic anisotropy, magnetization process, magnetoimpedance, ferromagnetic resonance, magnetic field sensors, microwave absorption

## Abstract

The ferromagnetic resonance (FMR) in the frequency range of 0.5 to 12.5 GHz has been investigated as a function of external magnetic field for rapidly quenched Fe_3_Co_67_Cr_3_Si_15_B_12_ amorphous ribbons with different features of the effective magnetic anisotropy. Three states of the ribbons were considered: as-quenched without any treatment; after relaxation annealing without stress at the temperature of 350 °C during 1 h; and after annealing under specific stress of 230 MPa at the temperature of 350 °C during 1 h. For FMR measurements, we adapted a technique previously proposed and tested for the case of microwires. Here, amorphous ribbons were studied using the sample holder based on a commercial SMA connector. On the basis of the measurements of the reflection coefficient S_11,_ the total impedance including its real and imaginary components was determined to be in the frequency range of 0.5 to 12.5 GHz. In order to confirm the validity of the proposed technique, FMR was also measured by the certified cavity perturbation technique using a commercial Bruker spectrometer operating at X-band frequency of 9.39 GHz. As part of the characterization of the ribbons used for microwave measurements, comparative analysis was performed of X-ray diffraction, optical microscopy, transmission electron microscopy, inductive magnetic hysteresis loops, vibrating sample magnetometry, magneto-optical Kerr effect (including magnetic domains) and magnetoimpedance data for of all samples.

## 1. Introduction

Rapidly quenched amorphous and nanocrystalline ribbons were the subject of intensive research in the last three decades [1,2,3]. Special interest in this kind of material can be explained by their extensive use in production techniques. It is reasonably stable and applicable in a wide variety of compositions, and low contamination can be ensured. In addition, the ribbon’s properties can be modified by the post preparation treatments according the particular application [3,4,5]. Among others, Co-based amorphous ribbons with close-to-zero magnetostriction were extensively studied with the focus on their applications to wound core in the field of transformers, working at frequency levels of hundreds Hz and magnetic sensors focused on the detection of small magnetic fields [6,7,8]. 

One of the most sensitive effects for small magnetic field sensor applications is magnetoimpedance (MI). The MI phenomenon consists of the change in the total impedance of a ferromagnetic conducting sample under application of an external magnetic field (H) and flow of a high frequency alternating current [4,9,10]. Makhotkin et al. reported the first ribbon-based alternating current magnetic sensor prototype in [11]. They introduced ribbon-based prototypes suitable for magnetic biosensing both in label-free and magnetic label detection regimes [12,13]. In the latter, the corrosion stability was very important and compositions with chromium or molybdenum [14,15,16] were considered to take account of the need for low or high corrosion. For the CoFeCrSiB composition, the best results were obtained after stress annealing, and a sensitivity with respect to the applied field of the order of 200%/Oe was achieved [17]. This was made possible by thorough previous studies on the annealing conditions: the temperature (T), annealing time (t) and specific load (σ) were carefully adjusted [18]. However, past research focused on the understanding of the dependence of magnetic properties, effective magnetic anisotropy and MI for rather high values of the induced magnetic anisotropy constant (K_u_) above 2000 erg/cm^3^. For the temperature of the stress annealing of 350 °C, there is only one study of MI with low specific load value under the following conditions: t = 1 h and σ = 210 MPa. This resulted in formation of transverse magnetic anisotropy with very low anisotropy distribution and high MI sensitivity of the order of 200%/Oe for the frequency of a flowing current (f) of about 10 MHz and samples of 10 cm in length [17]. However, other conditions were not investigated, and the transition from the longitudinal to the transverse anisotropy was not really understood.

As-quenched Co-based ribbons are characterized by the longitudinal magnetic anisotropy with the in-plane magnetic anisotropy axis along the long side of the ribbon. Relaxation annealing at temperatures below the crystallization temperature of the particular alloy results in the reduction of the level of the as-quenched stresses but does not change the type of the magnetic anisotropy, which continues to be longitudinal. Stress annealing with a negative magnetic anisotropy constant results in formation of the transverse uniaxial magnetic anisotropy, with the induced anisotropy axis oriented along the width of the ribbon and, again, the reduction of the level of the as-quenched stresses [19,20]. Such a transition (from the longitudinal to transverse effective anisotropy) can be studied in terms of the MI effect. Although the concept of using MI as an “instrument” was proposed during the first years of MI studies, the number of systematic MI works for the precise evaluation of anisotropy features is still limited.

The MI phenomenon has been understood in the context of classical electrodynamics and is well explained on the basis of the dependence of skin penetration depth (δ) on the dynamic magnetic permeability (μ) of a ferromagnetic conductor. That is, high frequency impedance depends on the skin penetration depth, which in turn depends on the magnetic permeability and applied magnetic field: Z = Z(δ(f,μ(H)) [1,2,3,4]. There have also been special studies related to the connection between MI and another well-known high frequency phenomenon—ferromagnetic resonance (FMR). FMR is the resonant absorption of microwave radiation in magnetic material [21,22]. Yelon et al. proposed the point of view that calculations of magnetoimpedance and of ferromagnetic resonance response are rigorously equivalent in the case of a plate or ribbon [23]. Experimental analysis included an evaluation of the changes in the attenuation coefficient of a coaxial line, having a NiFeMo wire as the central conductor for the frequency range of 100 to 6000 MHz. However, the number of studies of both MI and FMR remain very limited. Among other reasons, broadband measurement techniques were not widely available. They have become more accessible and applied to very different types of soft magnetic materials only in the last one and half decade [24,25,26,27]. For example, El Kammouni et al. studied FMR of amorphous microwires using a network analyzer (Agilent E8362B) in the frequency range of up to 12 GHz at a constant incident power of −10 dBm using a commercial SMA connector where the inner pin had been removed in order to avoid radiation effects [28]. The same technique was employed by Kurlyandskaya et al. for the case of CuBe/FeCoNi electroplated wires in the frequency range of up to 14 GHz [29]. In the latter, both FMR and MI measurements were presented.

In this present work, we proposed to design and test the broadband measurement technique based on the employment of coaxial waveguide, and a sample holder based on a commercial SMA connector. The reflection coefficient S_11_ measurements allowed accurate definition of the total impedance variation in the frequency range of 0.5 to 12.5 GHz. Special attention was paid on the selection and detailed characterization of the model samples prepared that were based on a single batch Fe_3_Co_67_Cr_3_Si_15_B_12_ amorphous ribbon. The amorphous structure was studied, and the static and dynamic magnetic properties of the ribbons with different features of effective magnetic anisotropy near the state of transition from longitudinal to a transverse magnetic anisotropy were comparatively analyzed using different techniques prior to the microwave tests.

## 2. Materials and Methods

Amorphous ribbons with nominal composition of Fe_3_Co_67_Cr_3_Si_15_B_12_ were prepared by rapid quenching onto a Cu drum. The length of one batch was as much as several meters with very close parameters of the width and thickness over the whole batch. According to the literature [3,15], the ribbons of such composition have very small negative saturation magnetostriction constant (λ_S_~10^−7^) and therefore they can be used for stress annealing and formation of the transverse magnetic anisotropy suitable for MI applications [13,19,30,31,32]. Apart from the as-quenched samples in the initial state (AP), the samples that were considered were obtained by the relaxation annealing (AN) at 350 °C during 1 h without load and stress annealing (SA) during 1 h and specific load of 230 MPa.

Selected composition differs from the widely studied Fe_4_Co_69_Si_15_B_12_ [33] material by the presence of chromium making it very stable in special environmental conditions including chemically active biofluids [6]. Fe_3_Co_67_Cr_3_Si_15_B_12_ ribbons have a saturation magnetization M_s_ = 365 G, and a Curie temperature of 160 °C which is quite low in comparison with their crystallization temperature of 570 °C. This means that selected heat treatments at 350 °C were performed at the temperature being approximately 0.6 of the crystallization temperature. For the majority of rapidly quenched amorphous alloys, such heat treatments do not change the amorphous structure of the material [1,34,35]. For example, Dmitrieva et al. [33] had studied the Fe_5_Co_72_Si_15_B_8_ alloy in the initial state, after relaxation or stress annealing. According to magnetic measurements and TEM data analysis, all materials were amorphous but the change of the structure was observed after annealing at 430 °C. The Fe_5_Co_72_Si_15_B_8_ alloy has a much lower crystallization temperature of 418 °C in comparison with that of Fe_3_Co_67_Cr_3_Si_15_B_12_, and a corresponding ratio of 1.02 between the temperature of the heat treatment and crystallization temperature.

The temperature and time of the annealing were selected on the basis of previous studies [3,17,18]. However, the specific load was modified, namely increased up to σ = 230 MPa in comparison with data in [18]. The following arguments were used for the selected conditions. Dmitrieva et al. had shown linear dependence of Ku(σ) for the specific load range 400–1400 MPa. The only specific load in the interval 0–400 MPa was 210 MPa reported by Kurlyandskaya et al. [17]. However, this was following the linear dependence of Ku(σ) with a somewhat lower regression. We therefore decided to slightly increase the specific load in the present study up to σ = 230 MPa in keeping with the joint data analysis reported in [17,18]. Heat treatments (both AN and SA) were done in the self-made vertical furnace with a thermocouple temperature control using a calibrated system.

**Figure 1:**
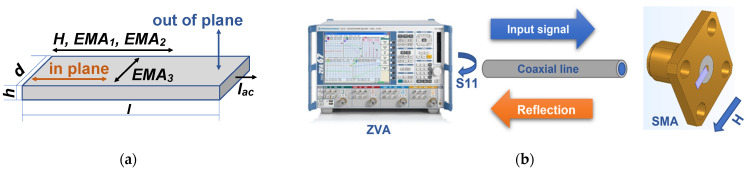
(**a**) Schematic description of amorphous ribbon sample with dimensions l × d × h: H is the external magnetic field applied during magnetic and GMI measurements, however for FMR measurements in the resonance cavity both in-plane and out-of-plane orientations of the external magnetic field were used; EMA_1_ corresponds to the easy magnetization axis of the sample in initial state; EMA_2_ corresponds to the easy magnetization axis of the sample after relaxation annealing; and EMA_3_ corresponds to the easy magnetization axis of the sample after TMO. (**b**) The scheme of the broadband microwave measurements using ZVA VNA for the coaxial position of a sample; H is an external magnetic field.

The widths and surface features of the ribbons from both sides (the free and weal sides) were studied by optical microscopy (Nicon L-UEPI microscope, Boston Industries, Inc., MA). The geometrical parameters of the samples were defined as follows: width—d, thickness—h and length—l (Figure 1).

The amorphous state of the samples in all conditions was checked by X-ray diffraction technique. These studies were performed by operating the DISCOVER D8 diffractometer (Bruker, Leiderdorp, The Netherlands) at 40 kV and 40 mA, using Cu-Kα radiation (wavelength of 1.5418 Å), a graphite monochromator and a scintillation detector. Ribbons were cut into pieces of about 1 cm and placed onto zero signal Si plate.

In addition, the ribbons of all types were studied by transmission electron microscopy using JEM 200CX electron microscope (JEOL, Freising, Germany). The samples for TEM analysis were prepared by electropolishing with H_3_PO_4_ + CrO_3_ fresh electrolyte. Both the bright field image for revelation of the microstructural features and the microdiffraction patterns were collected.

All magnetic and microwave measurements were made at room temperature. Magnetic measurements of the hysteresis loops M(H) were carried out by both a conventional inductive system and a vibrating sample magnetometer (VSM, Lake Shore 7404, Westerville, OH, USA) in the up to ±1.8 kOe field range. The inductive hysteresis loops were measured by applying a uniform external magnetic field in the plane of the ribbons of 45 mm length, i.e., the same length as the ones used for MI measurements (Figure 1). However, as FMR studies in both systems employed were made for short samples of 5 mm length, apart from the inductive hysteresis loops, VSM data were also collected and analyzed for samples of 5 mm length. In the VSM case, both in-plane and out-of-plane (Figure 1a) were carried out. In addition, magneto-optical Kerr effect (MOKE) was employed for magnetic measurements and observation of magnetic domains. The hysteresis loops were also measured by Kerr microscopy (Evico, Dresden, Germany) by plotting the average image intensity as a function of magnetic field.

The magnetoimpedance measurements were performed using a “microstrip” line with 50 Ohm characteristic impedance. The electrical contacts were made by a conductive silver paint for proper installation of the ribbon into microwave holder. The external magnetic field of up to H_max_ ± 110 Oe was created by a pair of Helmholtz coils. The total impedance (Z) was calculated from the reflection coefficient S_11_, after proper calibration and mathematical subtraction of the microwave fixture contributions. S_11_(H) values were measured by 4294A (Agilent/Keysight Technologies, Santa Rosa, CA, USA), using an output power of 0 dB. This means that the amplitude of the excitation current across the sample was about 10 mA. An external magnetic field was applied in-plane of the ribbon along the ribbon axis (long side of the ribbon) and in the same direction as the flowing current. The configuration of the longitudinal MI employed was the same configuration as for FMR: the radio frequency field created by the alternating current was perpendicular to the direction of the applied field. For the convenience of comparison of experimental results with the data reported by other researchers, the following MI ratio was used:

ΔZ/Z = 100% · (Z(H) − Z(H_max_))/Z(H_max_)). (1)

In addition, the sensitivity with respect to the constant applied field was defined as follows:

Δ(ΔZ/Z)(H)= (ΔZ/Z (H1) − ΔZ/Z (H_2_))/|H_1_ − H_2_|, (2)

where H_1_ is the smallest and H_2_ is the biggest values of the applied field for the interval of linear dependence of ΔZ/Z (H), i.e., the interval for which the sensitivity was calculated.

Ferromagnetic resonance measurements at microwave frequency f = 9.39 GHz were carried out on an ELEXSYS 500 Bruker spectrometer (Bruker, Tampa, FL, USA) operating by standard cavity perturbation technique protocol [22]. Both in-plane and out-of-plane configurations of the application of the external magnetic field were used. The maximum field value intensity was as high as 4.0 kOe (sufficient for magnetic saturation of the ribbons). 

In previous works [24,36], we proposed a way to measure the microwave properties of microwires that are electroplated and rapidly quenched wires in the coaxial line. The test fixtures, based on the SMA-holder for 1–15 GHz range, were designed and tested (for the cylindrical configuration). All test fixtures have individual characteristics such as terminal layout, dielectric constant of insulator, ground plate and so on. However, each one can be matched to the coaxial line with 50-Ohm characteristic impedance according to the established calibration process by using open, short and load reference terminations connected to the test port. Each of the terminations is measured, and the obtained impedance values of these reference terminations are analyzed in the form of vector impedance coordinates and a Smith chart. Compensation procedures allow the elimination of errors corresponding to test fixture contributions and to electrical length. The latter compensation eliminates measurement errors due to phase shift in the coaxial section [36].

Here we describe the possibility of adapting a previously designed system for precise measurements of the samples in the shape of prisms (amorphous ribbons) in a coaxial waveguide. The microwave properties of the ferromagnetic conducting ribbons were measured by the system based on a ZVA-67 vector network analyzer (VNA) (Rohde & Schwarz, Munich, Germany) using the one port method (Figure 1b). The electromagnet connected to the power supply TDK-Lambda GEN100-50 was capable of creating an external magnetic field with an intensity of up to 13 kOe. However, as amorphous ribbons are soft ferromagnets, the maximum value of the external magnetic field (sufficient for the ribbons’ magnetic saturation) was as high as 4.0 kOe. The power output of the signal of the ZVA analyzer was set at a level of 0.1 mW, based on the maximum signal-to-noise ratio criterion. In addition, at this level of power, the amplitude values of the intensity of an alternating magnetic field are significantly smaller than the value of the intensity of a field generated by a permanent magnet. Since no difference was observed between the positive and negative branches of measurements, the graphs discussed here present the values obtained in the positive field interval of 0.0–4.0 kOe only.

The measured samples were placed onto a holder made of a commercial connector SMA S-2454 (Mouser Electronics, Inc., Mansfield, TX, USA) connected to a coaxial cable (Pasternack PE3C0752) via a BN533795 adapter. The length of the samples of the microwires was chosen based on results of modeling. Since a ZVA-67 vector analyzer measures the amplitude and phase of the reflected signal as well, the value of the parameter S_11_ can be compensated for the length k of the adapter with the holder. Full one-port calibration was used to compensate the coaxial cable [24,36]. Although the range of the measurements using SMA in combination with VNA is 0.1 to 15 GHz, after careful modeling of the complete coaxial waveguide parameters with specific geometry used by High-Frequency Structure Simulator software, the appropriate frequency range was considered to be 0.5–12.5 GHz.

The reflection coefficient S_11_ values were measured as a function of the external magnetic field and frequency. These data can be used to estimate such parameters as microwave absorption of ferromagnetic samples. The next step was to determine the impedance of the sample Z with both real and imaginary parts. This required the compensation of the parameter S_11_ for the distance k:

(3) $$S_{11}^{、}={\;S}_{11}\times e^{i2\beta k}$$

where $S_{11}^{、}$ is the value of the parameter of the scattering matrix in the plane of the sample; $S_{11}$ is the value of the parameter of the scattering matrix in the calibration plane; β = 2π/λ is the wavenumber; λ is the wavelength; and k is the electrical distance of the coaxial transmission line. The sample impedance can therefore be determined as follows:

(4) $$Z=Z_{0}\times \frac{1+S_{11}^{、}}{1-S_{11}^{、}}$$

where Z_0_ = 50 Ohm is the impedance of the coaxial line. The change in the reactive part of the impedance ΔX(f, H) = X(f, H) − X(f, 0) and loss resistance ΔR(f, H) = R(f, H) − R(f, 0) under application of an external constant magnetic field were also taken into account for the analysis of the observed resonance phenomena.

## 3. Results and Discussion

**Figure 2:**
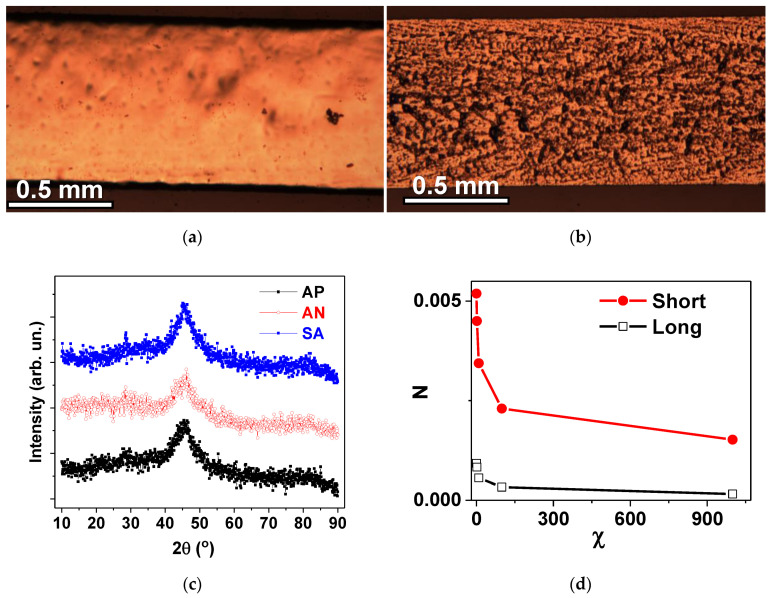
General view of the surface of amorphous ribbons obtained by optical microscopy: free side (**a**) and weal side (**b**) of the Fe_3_Co_67_Cr_3_Si_15_B_12_ amorphous ribbon. (**c**) XRD spectra for all types of the samples. (**d**) Demagnetizing factors calculated for selected values of magnetic susceptibility χ and two different lengths of the samples: l = 5 mm for “short” and l = 45 mm for “long” samples.

Table 1 summarizes the data on the states and types of treatments applied to the samples, and collects selected magnetic parameters. In a first step, the structure and geometrical parameters were defined. Figure 2a,b show the general view of the surface of the ribbons from both sides. The surface features were not changed during the heat treatments and the samples did not show any measurable elongation for the conditions under consideration. One can see that they have quite uniform surface features and well-defined width which was measured in the optical microscope after calibration. The width of the ribbons was 0.80 ± 0.02 mm and their thickness was 0.24 ± 0.01 μm.

The XRD analysis summary is given in Figure 2b. All types of the ribbons presented a clear amorphous structure and very broad diffraction peaks, 2θ(°) in a range of around 43–46° (Figure 2c), confirming the absence of long range ordering and crystalline phases in all cases under consideration.

**Figure 3:**
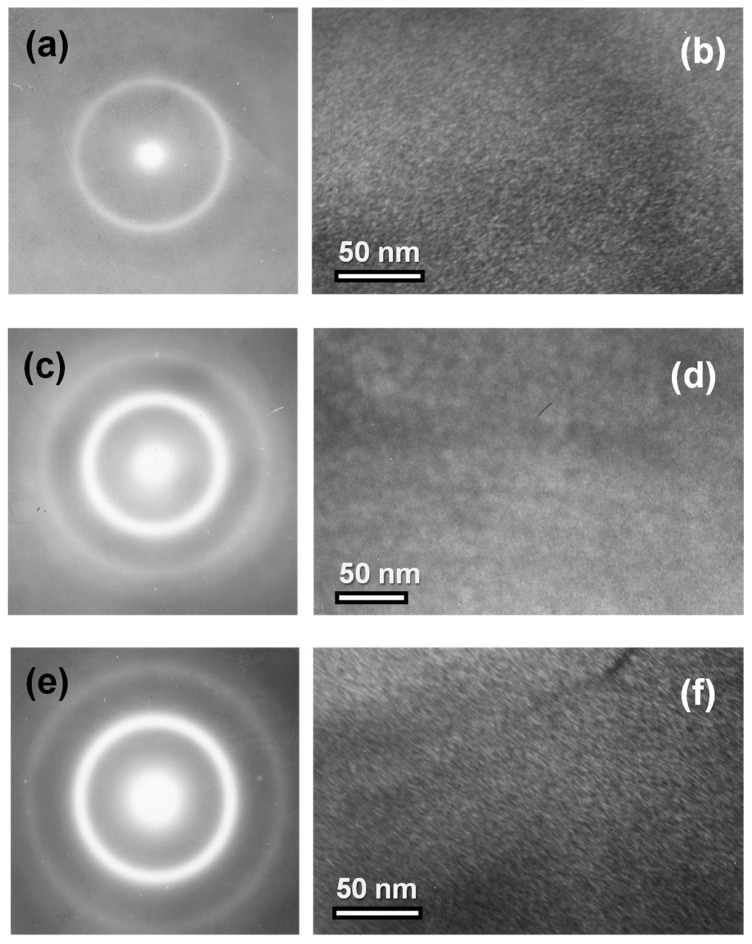
Microdiffraction patterns (**a**,**c**,**d**) and electron micrographs (**b**,**d**,**e**) for Fe_3_Co_67_Cr_3_Si_15_B_12_ rapidly-quenched ribbons in following states: AP (**a**,**b**); AN (**c**,**d**); and SA (**e**,**f**) (see also Table 1).

Such a result was highly expected for the ribbons of this composition [17] as the selected specific load value was very small. In addition, it was noted that mechanical properties of the samples were not visibly changed. The absence of significant changes (such as crystallization) in the amorphous structure was also confirmed by the analysis of TEM data. Figure 3 shows electron diffraction patterns and electron micrographs (×20,000) for different samples of the amorphous Fe_3_Co_67_Cr_3_Si_15_B_12_ alloy in all states under consideration: AP, AN and SA.

According to electron diffraction data, the samples of Fe_3_Co_67_Cr_3_Si_15_B_12_ alloy in all states are amorphous as they have no precipitates of crystalline phases. According to the existing literature, structural inhomogeneities can be formed in an amorphous alloy upon annealing at high temperatures following the rule that the higher the temperature, the higher the content of formed inhomogeneities. The inhomogeneities can have different chemical composition, size and features of a short-range order. It was previously shown that the prepared amorphous ribbons have less uniform structure in comparison with ribbons after stress annealing at a temperature of about 300 °C. The non-stoichiometry in the local composition and the tendency for atomic chemical ordering can result in the formation of clusters that are not considered as the first stage of crystallization but rather can increase the stability of the amorphous material [33]. 

Model considerations were given to the role of the micro-inhomogeneities contributing to the structural changes under uniaxial tensile stresses. Clusters may become anisotropic in shape and after cooling down due to difference in the thermal expansion coefficients of the amorphous matrix and clusters. Moreover, the magnetoelastic anisotropy at the interface between the matrix and the clusters may orient the magnetization in the transverse direction, i.e., along the short side of the ribbon [35]. However, the objectives of the present work do not include such a fundamental question as the nature of the induced magnetic anisotropy in the amorphous rapidly-quenched ferromagnets. Here, we use different structural and magnetic techniques for advanced characterization of the samples, which are prepared for rigorous testing of the microwave absorption technique proposed for broadband ferromagnetic resonance measurements. 

We now discuss static magnetic properties of the samples of different lengths. The size of the functional elements of rapidly-quenched ribbons can vary significantly from tens of cm for wound transformers to a few mm for sensitive element of the magnetic field sensors. The samples of 45 mm length (denominated as “long” samples) were prepared for magnetoimpedance testing. Their static magnetic hysteresis loops were measured using an inductive technique. Both VSM and FMR techniques required preparation of the “short” samples of 5 mm length. The length difference caused a change in the demagnetizing factor, affecting the value of the effective magnetic anisotropy on the ribbons of each particular geometric length. 

In order to take into account the difference in the geometry of the ribbons, we calculated the demagnetizing factors of the prisms with the corresponding geometrical parameters for selected values of magnetic susceptibility from 0 to 999. It is well known that demagnetizing factors (N) for the objects of such shapes can be calculated only approximately [37]. However, Chen et al. proposed a useful technique for calculations of demagnetizing factors of rectangular [38] prisms, which was employed for N value calculations for the “short” and “long” ribbons under consideration (Figure 2d). One can see that calculated demagnetizing factors are significantly higher in the case of the “short” ribbons for all considered magnetic susceptibility values.

**Figure 4:**
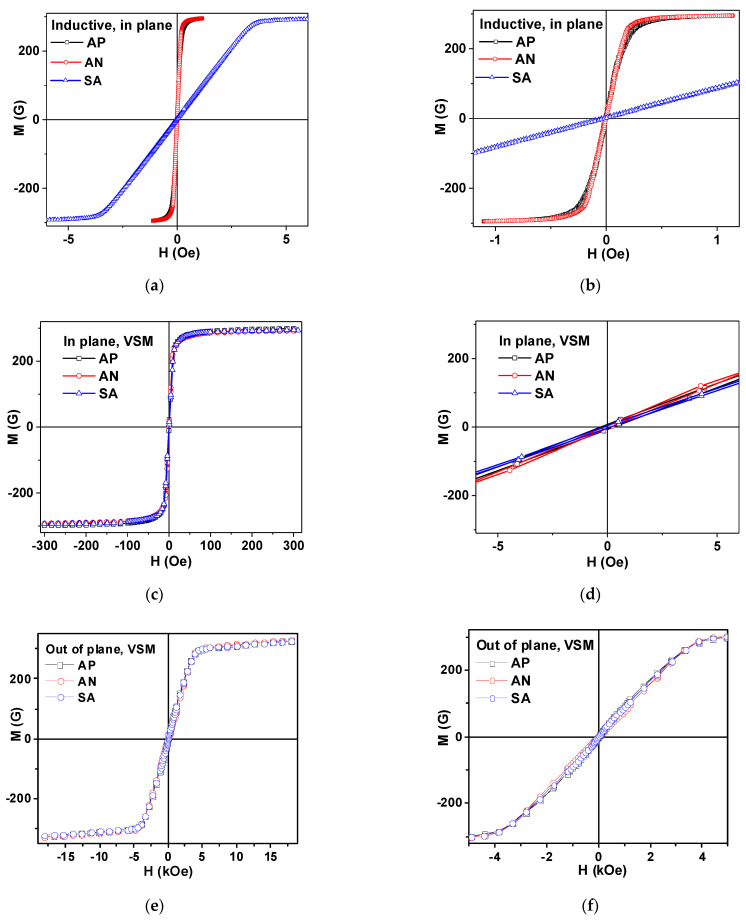
Magnetic hysteresis loops of amorphous Fe_3_Co_67_Cr_3_Si_15_B_12_ ribbons in different states, with AP, AN and SA measured by: inductive technique (**a**,**b**); by VSM in the plane of the sample (**c**,**d**); and out-of-plane of the sample (**e**,**f**). For inductive measurements, sample length was l = 45 mm and for VSM samples, the length was l = 5 mm.

Figure 4a,b show the results of the measurements of inductive hysteresis loops along the long side of the ribbons. One can clearly see that AP and AN ribbons have longitudinal magnetic anisotropy with very similar shaped M(H) loops and very small difference in the field range of 0.1 to 0.3 Oe; andthe sample after relaxation annealing shows a slightly faster approach to magnetic saturation. Both EMA_1_ and EMA_2_ are parallel to each other and to the long side of the ribbon. 

The small difference can be a result of the lower level of residual stresses in AN samples subjected to relaxation annealing treatment. The anisotropy of the SA ribbon is clearly transverse with EMA_3_ oriented parallel to the short side of the ribbon. The SA ribbon has small coercivity and shows linear M(H) dependence up to the anisotropy field H_a_. Using the familiar definition [3,31] of the induced magnetic anisotropy constant $K_{u}=1/2\cdot M_{s}H_{a}$, where M_s_ is the saturation magnetization, we calculated the *K_u_* value for the TMO sample (see also Table 1). The most telling result of the magneto-inductive measurements is the difference in effective magnetic anisotropy of AP, the AN ribbons having longitudinal and SA ribbons having transverse magnetic anisotropy with narrow anisotropy distribution, quite in agreement with previous studies with comparable values of the specific loads and thermal conditions. 

Figure 4c,d show the results of the measurements of VSM hysteresis loops for the magnetic field applied in the plane of the ribbons. Figure 3e,f show the results of the measurements of VSM hysteresis loops for the magnetic field applied out-of-plane of the ribbons. VSM M(H) loops for all sample types are very close to each other: all in-plane loops for AP, AN and SA ribbons are similar to each other and all out-of-plane AP, AN and SA magnetic hysteresis loops are very similar to each other. However, as expected, in-plane and out-of-plane loops are different. The remarkable difference between the loops shown in Figure 3a,c for the same types of samples but of different lengths can be understood by taking account of the demagnetizing factor N and demagnetizing fields related to the shape anisotropy (Figure 2d). The shape anisotropy contribution becomes dominating in the short samples, making the induced anisotropy contribution negligible. As the effective magnetic anisotropy features are crucial for magnetoimpedance responses [8,17,32], from the peculiarities of M(H) hysteresis loops one can immediately deduce that MI differences should be important for the long samples only.

**Figure 5:**
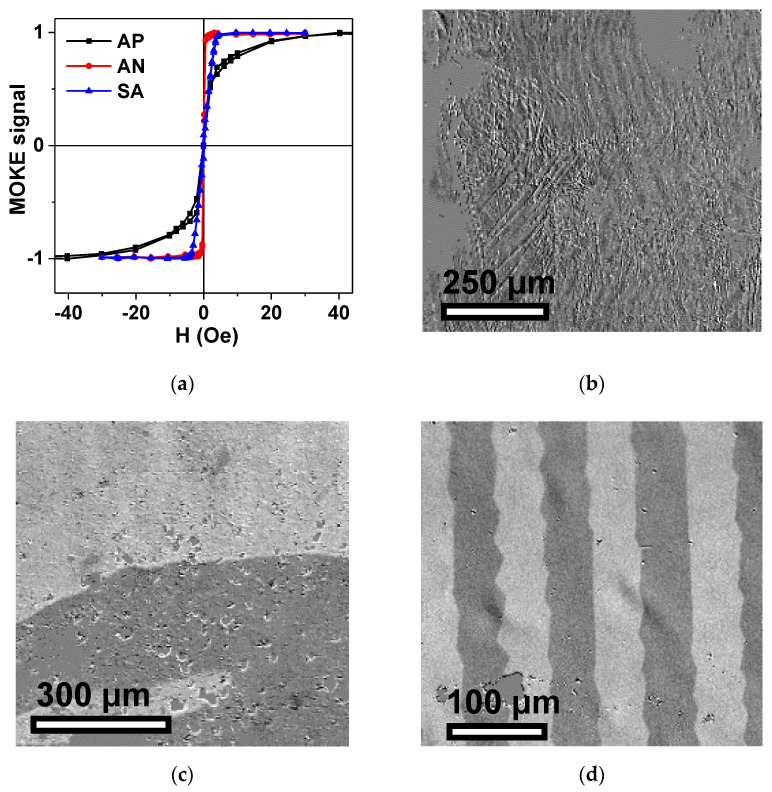
MOKE hysteresis loops for amorphous Fe_3_Co_67_Cr_3_Si_15_B_12_ ribbons in AP, AN and SA states measured for the external magnetic field applied in the plane of the ribbon and along the long side of the ribbon (**a**). Magnetic domain structure for amorphous Fe_3_Co_67_Cr_3_Si_15_B_12_ ribbons in states: AP (**b**), AN (**c**) and SA (**d**). External magnetic field is equal to zero. Ribbon´s long side is oriented in horizontal direction for all images.

For confirmation of the magnetic measurement data related to the volume properties of the Fe_3_Co_67_Cr_3_Si_15_B_12_ ribbons in AP, AN and SA states, MOKE investigations were also performed in order to ensure the complete characterization of the samples. Figure 5a shows the hysteresis loops measured in a Kerr microscope by plotting the average image intensity as a function of the external magnetic field applied along the long side of the ribbon in the ribbon plane, i.e., in the geometry most often used in the sensor applications (longitudinal magnetoimpedance geometry). One can clearly see a quite reasonable match between inductive hysteresis loop features (Figure 4a,b): AP and AN samples have longitudinal magnetic anisotropy, and SA sample transverse. One feature to mention is the difference between AP and AN behaviors in the intermediate fields, with a much lower approach to magnetic saturation state in the case of the AP ribbon. The observed difference is quite understandable if we take into account that MOKE collects information mainly from the surface of the sample. For the visible white light employed, the estimated penetration depth was about 30–35 nm. This means the information depth of half that range (the light has to return to surface for collection by the detector) was close to but below 20 nm [39]. 

The surface structure and surface anisotropy in as-quenched ribbons differ from the structure and anisotropy of the body of the sample. The thickness of the surface layer in which the most pronounced difference is observed depends on the technological parameters of the ribbon quenching, as well as on the composition of the ribbon [5,6,34]. The objective of the present study does not include complete evaluation of the anisotropy features of all Fe_3_Co_67_Cr_3_Si_15_B_12_ ribbons in AP, AN and SA states obtained from one batch. We only provide the characterization of different model samples for high frequency testing of the proposed microwave technique. Even so, MOKE, VSM and inductive measurements result are all in a good agreement with existing models and experimental reports. For more details, [5] can be referred to, where correlation between domain structure, surface anisotropy and high frequency magnetoimpedance were comparatively analyzed in CoFe-based melt-spun ribbons after Joule annealing.

Figure 5b–d show typical examples of the magnetic domain structure in zero external magnetic field, specifically after previous saturation in the positive external field of 100 Oe applied in the plane of the ribbon and along the long side of the ribbon. Observed magnetic domain structures are in very good agreement with the features of magnetic hysteresis loops obtained by the different techniques, and they are in accordance with the data of other researchers [40,41]. As before, in the AP state, the domain structure is irregular and the domain patterns are not oriented with respect to the ribbon axis. The surface domains in AP samples most probably differ from the body domains. After relaxation annealing and redistribution of the surface stresses, the magnetic domains revealed in the AN ribbons become wide domains with 180° domain walls typical for soft ferromagnetic material with uniaxial magnetic anisotropy and low level stresses. For the SA sample, different behavior occurs. At zero external field, a domain pattern is related to a stripe domain or V-line type [41], and the in-plane component of magnetization is oriented perpendicularly to the direction of the applied stress. Observed domains are bounded by 180° domain walls complicated by “zigzag” type refinements [42].

**Figure 6:**
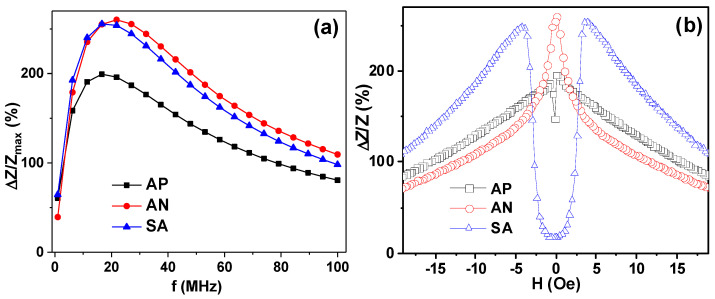
(**a**) Frequency dependence of the maximum MI ratio for amorphous Fe_3_Co_67_Cr_3_Si_15_B_12_ ribbons in AP, AN and SA states, measured in longitudinal MI configuration. (**b**) The applied field dependence of the MI ratio for the same samples with length l = 45 mm and f = 22 MHz.

The next step is the high frequency characterization of the obtained materials in the MHz interval of the frequencies. Figure 6 shows selected results of comparative studies of MI parameters for all the kinds of ribbons under consideration. The frequency dependences of the maximum MI ratio ΔZ/Z_max_(f) are consistent with predictions of classical electromagnetic theory for uniform electromagnetic conductor: maximum MI ratio increases, reaches the maximum and decreases afterwards [4,12,31,32].

For all frequencies above 1 MHz, the MI ratios of the AP ribbons have the lowest values. In the frequency range up to about 20 MHz, MI ratios of annealed and stress annealed samples are very close to each other, but above 20 MHz the sample after the relaxation annealing (AN) shows slightly higher ΔZ/Zmax values. However, both ΔZ/Z_max_ values (for AN and SA samples) are higher than the ΔZ/Z_max_ for the AP ribbon. This is to be expected as both AN and SA were subjected to relaxation heating and therefore they are characterized by a decrease in the level of frozen-in stresses. 

For the analysis of the shape of the ΔZ/Z(H) dependences, the 22 MHz frequency was selected being the closest frequency after reaching the maximum by each of the ΔZ/Z_max_(f) curves. The shapes of the ΔZ/Z(H) dependences are very telling—they are in great agreement with the M(H) hysteresis loops measured by inductive technique for the samples of the same lengths (Figure 3a,b). AP and AN samples are typical for longitudinal effective magnetic anisotropy, displaying as they do a “one peak” shape [6,17]. In the case of the AP ribbon in the close-to-zero field, an additional “valley” appears as manifestation of the contribution of the surface anisotropy in the sample with a high level of residual stresses [6]. However, the TMT sample with uniaxial transverse magnetic anisotropy shows a very well-defined “two peak” shape with maxima positions near the anisotropy fields.

Figure 4b shows that the SA amorphous ribbon is an excellent candidate for small magnetic field detection due to very high sensitivity with respect to applied field, and a reasonably low field (few Oe) required to reach the highest sensitivity. Calculation of the maximum sensitivity with respect to an applied magnetic field gives the following values: Δ(ΔZ/Z) = 9%/Oe (0.10 < H < 1.80 Oe) for AP; Δ(ΔZ/Z) = 50%/Oe (0.15 < H < 1.80 Oe) for AN; Δ(ΔZ/Z) = 140%/Oe (1.80 < H < 3.20 Oe) for SA. The field interval for linear dependence of ΔZ/Z(H) and the sensitivity rate are very good for creation of the small magnetic field sensitive elements with shifted work point. One of the advantages of the proposed SA material is the reduced lengths of the sensitive element showing such an advanced sensitivity. In many previous works, the lengths of the sensitive elements were approaching 10 cm [17]. 

For short samples there exists a limit of about 2 cm, below which the length plays a critically important role [15]. However, as the demagnetizing fields depend on all three geometrical parameters, the most correct way for evaluation is to use the demagnetizing factor [38], which still leaves the possibility of improving the functional properties of the ribbons by proper selection of widths and thickness. Comparative analysis of the obtained parameters shows that the SA ribbon has in-plane uniaxial magnetic anisotropy with very low anisotropy distribution, providing high dynamic magnetic permeability when the external magnetic field approaches the anisotropy field, and therefore very high MI sensitivity.

**Figure 7:**
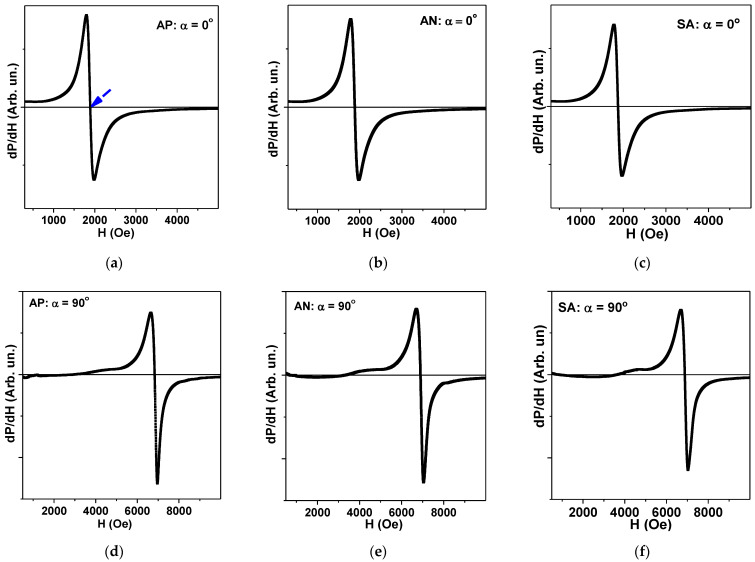
(**a**–**c**) Ferromagnetic resonance curves dP/dH of Fe_3_Co_67_Cr_3_Si_15_B_12_ amorphous ribbons in the different states AP, AN and AP measured in-plane, respectively, with α = 0°; and (**d**–**f**) out-of-plane of the samples, respectively, with α = 90°. Blue arrow (**a**) indicates the orientation of the FMR resonance field (H_res_) definition. All measurements were done for the microwave frequency f = 9.39 GHz and at room temperature.

For FMR measurement evaluation, we consider the shape of the sample to be a prism, as we do for the calculation of the demagnetizing factor. For selected geometrical parameters, thickness h is quite small in comparison with lengths and widths. In this case, all standard equations of FMR are applicable for the description of the microwave properties of the ferromagnetic sample in the shape of a thin plate [22,43,44,45]. The derivative of the microwave absorption power (P) with respect to applied magnetic field dP/dH was measured. Figure 7 shows the results of the resonant microwave absorption measurements in two requested thin plate configurations. The resonance field values, H_res_, were measured in-plane and out-of-plane at angle α= 0° for in-plane and α= 90° for out-of-plane configurations.

For uniaxial magnetic anisotropy where the symmetry axis is along the film normal, one can write:

(5) $${\left(\frac{\omega }{\gamma }\right)}^{2}=H(H+4\pi M_{\mathrm{eff}})\;(\alpha =90{}^{\circ})$$

(6) $${\left(\frac{\omega }{\gamma }\right)}^{}=H-4\pi M_{\mathrm{eff}}(\alpha =0{}^{\circ})$$

where 4πM_eff_ is effective magnetization, ω = 2πf is the angular frequency, and γ is the gyromagnetic ratio. Equations (5) and (6) can be used to determine effective magnetization values for all samples and compare them with the data of VSM measurements. We take the FMR resonance value to be an external field, at which the dP/dH response becomes zero when crossing the dP/dH = 0 axis between two maxima of dP/dH. 

Table 2 summarizes the main results of the FMR measurements by cavity perturbation technique and the analysis and comparison of the effective magnetization value 4πM_eff_ (found from common solution of Equations (5) and (6) with the saturation magnetization M_s_ values obtained by VSM).

Here we should make an additional remark about the accuracy of the determination of M_eff_ and M_s_ values. Although they are in reasonable agreement, as often happens [22,46], M_eff_ tends to be lower due to the contribution of magnetoelastic anisotropy and internal stresses even when perpendicular magnetic anisotropy is negligible. The origin of the experimental error of the saturation magnetization obtained from VSM measurements comes from the need to recalculate emu/g units into Gauss using a parameter such as density. The determination of the correct values of the density of rapidly quenched amorphous ribbons was recently the subject of very special comparative analysis by Parsons et al. [46]. Their arguments and conclusions together with the data existing in the literature gave us the basis to consider the density of the ribbons under consideration to be ρ = 7.1 ± 0.2 g/cm^3^. For FMR measurements, the main uncertainty comes from the width of the resonance line of amorphous ribbon. For all samples measured by the cavity perturbation technique, these values were close to 0.28 ± 0.02 kOe.

**Figure 8:**
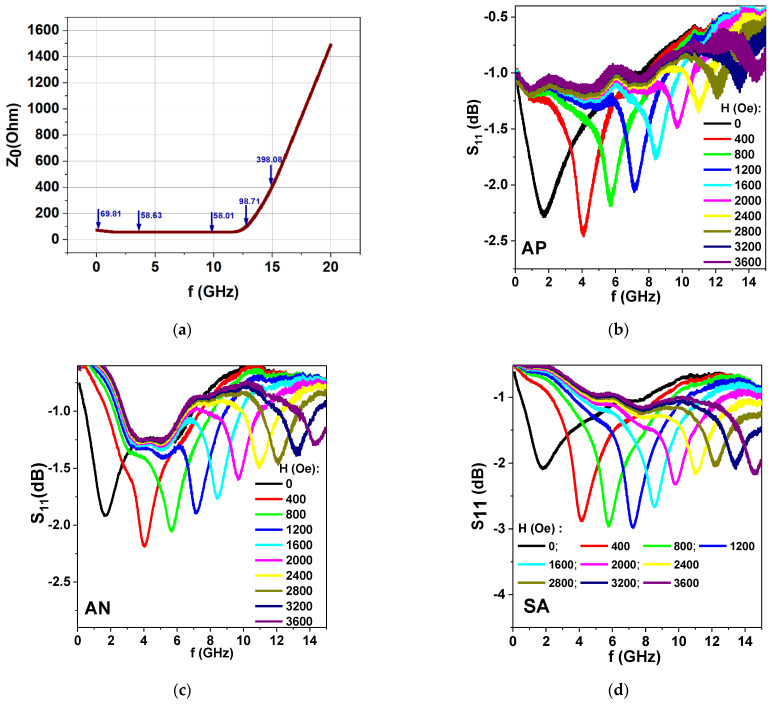
(**a**) Calculation of characteristic impedance Z_0_ (values indicated by blue arrows) of a waveguide consisting of a coaxial line and a thick planar sample. Blue arrows show the simulation results for the approximation to the 50 Ohm matching waveguide characteristic impedance (in the frequency range of 0.5 to 12.5 GHz). (**b**–**d**) S_11_ parameters measured in the broadband regime for Fe_3_Co_67_Cr_3_Si_15_B_12_ amorphous ribbons in different states AP, AN and SA for the external magnetic field applied in the plane of the ribbons. The measurements were made for different values of the external magnetic field.

As the first step of broadband microwave absorption measurements, we calculated a complete set of coaxial waveguide parameters with complex geometry in software HFSS (High-Frequency Structure Simulator). This was necessary in order to minimize the electromagnetic reflection and absorption along the waveguide. We considered the possibility of using SMA holders with a given geometry and planar ribbon sample (see Figure 1b). The method of conformal mapping keeps the value of the wave characteristic impedance constant (~50 Ohm) by correctly varying the geometrical parameters of the sample [47]. Simulation results show an approximation of the 50 Ohm matching waveguide characteristic impedance (Z_0_) to be in the frequency range of 0.5 to 12.5 GHz. As the second step, S_11_ parameters were measured by the standard procedure (Figure 8) using in- plane configuration.

For all samples under consideration, the frequency dependences S_11_(f) have well defined absorption minima corresponding to the ferromagnetic resonances, H_res_, in a particular field for each given frequency. The frequency of the resonant absorption depends on the value of the applied external field. In order to analyze the connection between the resonance field value and the frequency to obtain FMR, one can use the familiar Kittel’s resonance condition for magnetized planar sample [21]:

(7) $${\left(\frac{\omega }{\gamma }\right)}^{2}=(H_{\mathrm{res}}+H_{a})(H_{\mathrm{res}}+H_{a}+4\pi M_{s})$$

where ω = 2πf is the resonance angular frequency of the microwave electromagnetic field and γ/2π = 2.8 × 10^6^ Hz/Oe is the gyromagnetic ratio.

**Figure 9:**
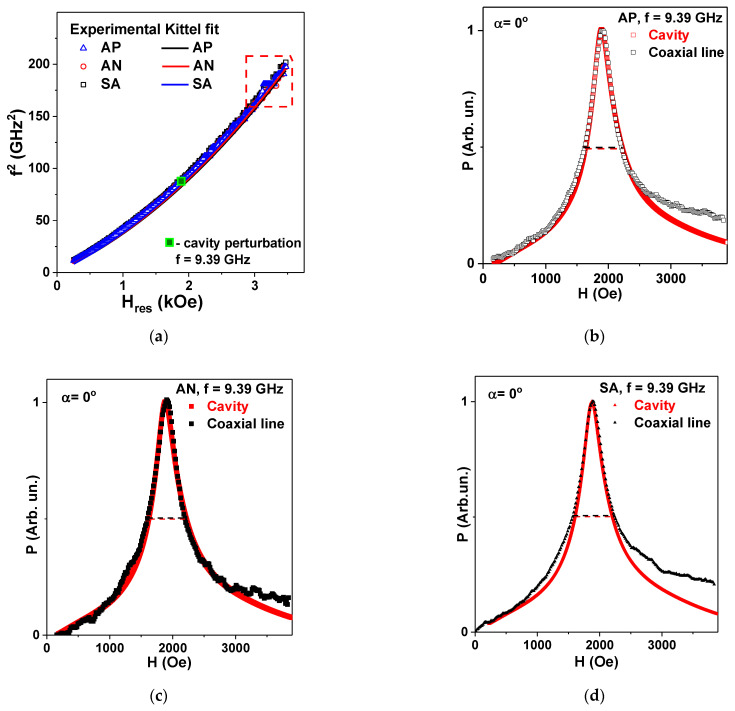
Microwave absorption measurements using a complete coaxial waveguide for Fe_3_Co_67_Cr_3_Si_15_B_12_ amorphous ribbons in different states AP, AN and SA. Experimental results and Kittel´s fit. (**a**) Dashed rectangle indicates the frequency range where the measurements are still possible but less precise as the approximation to the 50 Ohm matching waveguide characteristic impedance (Z_0_) deviates. (**b**–**d**) Direct comparison of the FMR resonance lines obtained for the frequency f = 9.39 GHz both by the cavity perturbation technique and using the coaxial waveguide for all ribbon types. Red and black dashed lines indicate the width of the FMR resonances.

Figure 9a shows experimental f^2^(H_res_) dependences for all ribbon types and corresponding fits of Equation (6) using experimental parameters obtained from magnetic measurements. Both experimental curves of all types and the fitted curves show quite similar evolution, indicating the high quality of the proposed method for evaluation of broadband properties. 

Here we draw attention to the fact that, according to HFSS simulation, the appropriate measurements range was 0.5 to 12.5 GHz. Although Figure 8a represents the f^2^ range up to about 14 GHz (f^2^ ≈ 200 GHz^2^), the data above 12.5 GHz become less precise. The dashed rectangular frame indicates this range of deviations. In addition, the excellent match for cavity perturbation technique data is also shown by indicating the position for the cavity perturbation measurement, which appears to be very close to the broadband measurements for corresponding the frequency.

Figure 9b–d show experimental P(H) dependences for all types of Fe_3_Co_67_Cr_3_Si_15_B_12_ amorphous ribbons measured by both the standard cavity perturbation technique and the coaxial waveguide. As the original measurements (done by the commercial spectrometer) were made in dP/dH mode, in order to perform such a comparison, the initial signals dP/dH were mathematically integrated with respect to the external magnetic applied field and the maximum of the absorption power intensity was normalized to one unit. 

For proper comparison, the reflection coefficient S_11_ measured by the ZVA VNA analyzer for the calibration plane was recalculated in accordance with the method proposed in [29,36], and the changes in the real and imaginary parts of the impedance (ΔR and ΔX) were obtained. For comparison between the two different microwave techniques, the ΔR part was employed. Figure 9b–d show the broadband measurements data for all ribbon types. We used the normalized value of the ΔR variation for the field dependence for the purposes of direct comparison with the cavity perturbation results. 

One can see very good agreement between the FMR (H_res_) measured by the two microwave techniques. Not only are the positions of the maximum values of the microwave absorption quite similar, but this also applies to the width of the FMR lines. In rigorous evaluation terms, we note that the width of the resonance line is better defined for the cavity perturbation case. In the case of the coaxial waveguide, the main problem is the correct determination of the base line. Even so, Figure 9 convincingly confirms the results of the measurements at f = 9.39 GHz. Strictly speaking, the comparison for Kittel´s conditions was made for one resonance frequency only and this is a disadvantage. However, very good agreement between the experimental results and fit of the Equation (7) is obtained, and the proposed measuring technique seems to be well adapted to the high frequency broadband characterization of the amorphous ribbons. 

It is necessary to emphasize that depending on the selected lengths of the Fe_3_Co_67_Cr_3_Si_15_B_12_ amorphous ribbons, the obtained materials can be used for high frequency applications in different frequency ranges. The independence of the FMR responses of short ribbons on the anisotropy features under consideration is also very interesting. It indicates the flexibility of the material and its potential for avoiding very strict protocols of additional heat treatments, saving time and energy in the case of some particular applications. 

One of the tendencies of the development of present-day microdevices and microsystems is an extension of the operating frequency range. This feature requires further development of the characterization techniques for different kinds of magnetically soft ferromagnets [5,31,36,48]. Here we proposed a simple way to measure the broadband FMR characteristics for amorphous ribbons. However, it can also be used for nanocrystalline materials and foils of rectangular shape.

## 4. Conclusions

Produced from one batch of the material, rapidly quenched Fe_3_Co_67_Cr_3_Si_15_B_12_ amorphous ribbons were prepared and designed in order to obtain model materials with different features of effective magnetic anisotropy for microwave tests. The following states were considered: (AP)—as-quenched without any treatment; (AN)—after relaxation annealing without stress at the temperature of 350 °C during 1 h; and (SA)—after annealing under specific stress of 200 MPa at the temperature of 350 °C during 1 h. In accordance with XRD and TEM studies, all materials were in amorphous state.

Magnetic measurements of the long ribbons (45 mm) using inductive technique and MOKE revealed some differences in the effective magnetic anisotropy features, namely: AP and AN samples had well defined longitudinal effective magnetic anisotropy. Stress annealed ribbons had well-defined transverse effective anisotropy with low dispersion of the local magnetic anisotropy axes. SA ribbons showed excellent magnetoimpedance properties with very high sensitivity above 130%/Oe for a frequency of about 20 MHz, which is very convenient for sensor applications. At the same time, VSM measurements of the short ribbons (5 mm) showed similarity of their magnetic characteristics due to strong contribution of the shape anisotropy.

Following this thorough characterization, the designed materials were used for microwave absorption measurements. For FMR tests of the short (5 mm) samples, we proposed, designed and tested a broadband measurement technique based on the employment of coaxial waveguide and sample holder established from a commercial SMA connector. Measurements of the reflection coefficient S_11_ allowed the accurate determination of the total impedance variation in the frequency range of 0.5 to 12.5 GHz. The validity of the proposed technique was also confirmed by the measurements using the traditional cavity perturbation technique (spectrometer operating at X-band frequency of 9.39 GHz).

## Figures and Tables

**Table 1:** Description of the types and states of rapidly quenched Fe_3_Co_67_Cr_3_Si_15_B_12_ ribbons and conditions of their thermal treatments. Selected parameters, obtained for long samples of 45 mm: K_u_—effective magnetic anisotropy constant, H_c_—coercivity, H_a_—magnetic anisotropy field and M_s_—saturation magnetization.

Sample	Description	K_u_, erg/cm^3^	H_c_, Oe	H_a_, Oe	M_s,_ Gs
AP	As-prepared	0	0.1	0.1	300
AN	Annealed at 350 °C without stress during 1 h	0	0.1	0.1	300
SA	Stress annealing at 350 °C without stress during 1 h for specific stress of 230 MPa	500	0.2	3.3	300

**Table 2:** Selected parameters of FMR measured for Fe_3_Co_67_Cr_3_Si_15_B_12_ amorphous ribbons in different states AP, AN and TMT for the frequency f = 9.39 GHz. H_res_—FMR resonance field; α = 0°—in-plane configuration, α = 90°—out-of-plane configuration; M_eff_—effective magnetization obtained from FMR measurements and M_s_—saturation magnetization obtained from VSM measurements.

Sample	H_res_, kOe α = 0°	H_res_, kOe α = 90°	4πM_eff_, kOe	M_eff_, G	M_s,_ G
AP	1.89	6.9	3.7	300 ± 20	330 ± 20
AN	1.90	6.9	3.7	300 ± 20	330 ± 20
SA	1.88	6.9	3.7	300 ± 20	330± 20

## Data Availability

Data are available from the corresponding author on reasonable request.
